# Structural comparison of the rostra of two species of weevils coexisting on *Ailanthus altissima*: the response to ecological demands of egg deposition

**DOI:** 10.1186/s12862-021-01824-7

**Published:** 2021-05-28

**Authors:** Ganyu Zhang, Wenjuan Guo, Xiaoyi Wang, Qian Wang, Jin Cui, Junbao Wen

**Affiliations:** Beijing Key Laboratory for Forest Pest Control, Beijing Forestry University, Beijing, 100083 China

**Keywords:** *Eucryptorrhynchus scrobiculatus*, *Eucryptorrhynchus brandti*, *Ailanthus altissima*, Rosrta, Structure, Oviposition

## Abstract

**Background:**

Elongated rostra play an important role in the egg-laying of weevils, and its emergence plays a key role in the adaptive radiation of weevils. *Eucryptorrhynchus scrobiculatus* Motschulsky and *E. brandti* Harold co-occur on the same only host *Ailanthus altissima*, while their oviposition sites are different. In order to understand the adaptation between the rostra of the two weevils and their oviposition sites, the structural differentiation of the rostra in *E. scrobiculatus* and *E. brandti* was compared.

**Results:**

The present study reveals that: (1) The rostra length of *E. scrobiculatus* and *E. brandti* was found to be correlated with body size, larger weevils have a correspondingly longer rostrum. The increase of rostra length may be a byproduct of larger weevils. (2) There were significant differences in the external shape of the two rostra, especially the shape of the mandibles of the mouthparts at the apex of the rostra used to excavate an oviposition cavity. (3) There was no difference in the size of the abductor muscles that control the extension of the mandibles, but there were significant differences in the size of the adductor muscles that control the contraction of the mandibles.

**Conclusions:**

These structural differences reflect the functional potential ovipositional tactics of rostra, which is considered to be a response to the ecological demands of egg deposition, and also provide new insights into the coexistence of two weevil species in the same host *A. altissima*.

**Supplementary Information:**

The online version contains supplementary material available at 10.1186/s12862-021-01824-7.

## Background

Weevils are a type of beetles belonging to the superfamily Curculionidea. They are one of the most diverse family-level groups of extant organisms, with approximately 60,000 described species [1]. The apical evolution of weevils is a key innovation that enables the group to feed and lay eggs in almost all plant tissues, resulting in different life histories and great diversity. They exhibit a vastly extended rostrum from the forehead, particularly in the females [2–4]. The tip or apex of the rostrum is called mouthpart, it is used to excavate an oviposition cavity by chewing a narrow opening into the tissues of the host. The use of the rostrum in the preparation of oviposition sites is considered to be a key adaptation measure, which helps to bypass the physical defense of plants (shells, spines), avoid the drying of larvae, and facilitate the initiation and maintenance of attachment to the host [1].

The variation of rostra type is considered to be the product of ecological action, and the rostrum size of each species is critical to their lifestyle and survival patterns. For example, the species of Curculio have a wide range of attacks on host plants. It is speculated that it is caused by the ecological morphological adaptation of the oviposition sites (host plant seeds). The size of host seeds is the cause of morphological changes in the size of the rostrum [2]. The length of the rostrum of different populations of the *Curculio camelliae* females is related to the thickness of the camellia pericarp [4, 5]. Morphological analysis is an indispensable tool in the research process of ecologists and evolutionary biologists, this method can try to link the structure and function of organisms with the characteristics of their connected environments [6]. For structure-function relationships, morphological studies can reveal selectivity factors in the environment and the limitations of phenotype responses to these factors. Compared with males, mouthparts and mandibles of adult females of *Doubledaya bucculenta* Lewis (Coleoptera: Erotylidae: Languriinae) show obvious directional asymmetry. This structure helps females to lay eggs in internodes of the bamboo and improve their adaptability to the offspring [7]. Gertha wilhelm showed that the rostrum of females and males of *Rhopalapion longirostre* (Coleoptera, Brentidae, Apioninae) has dimorphism. *Rhopalapion longirostre* females use their rostra to excavate egg channels in the bud. The length of the female’s rostrum is twice that of the male, and the surface of the female’s rostrum is smoother than that of the male [8]. Bland compared the mouthparts and sensors of *Hypera postica* and *H.brunneipennis*, which found that the types and numbers of cone sensors were the same at the endpoint, but the shape and size of the mandible were different between the two species [9, 10].

*Ailanthus altissima* (Mill). Swingle, also called the tree of heaven, is a common afforestation tree and street tree in China but is an invasive species that is not conducive to the growth of native plants in the United States [11, 12]. It is a species native to China and North Vietnam [13, 14]. However, in recent years, due to the damage of two major pests, *Eucryptorrhynchus scrobiculatus* Motschulsky and *E. brandti* Harold, this tree species has been severely damaged. According to statistics, affected by two kinds of weevils, the damage rate of *A. altissima* in Huaibei area of China was 80%, the damage rate of *A. altissima* in Ningxia area was more than 15%. The more serious was that the damage rate of *A. altissima* in Aksu City, Xinjiang was as high as 99.4% [15–18]. When the damage was serious, the number of *E. scrobiculatus* and *E.brandti* on a tree can be up to more than 360. The larvae fed and cut off the tissue of their host plant caused the death of *A. altissima*, which posed a serious threat to the greening forests and farmland protection forest nets around the villages and towns dominated by *A. altissima*. Based on the above circumstances, the State Forestry Administration of China successively included the *E. scrobiculatus* and the *E .brandti* in the “National Forestry Hazardous and Pest List” in 2003 and 2013 [19].

*Eucryptorrhynchus scrobiculatus* Motschulsky and *E. brandti* Harold belong to Coleoptera, Curculionidae, Cryptorrhychinae, Eucryptorrhynchus [20, 21]. These two species only harm *A. altissima* and its variant *A. altissima* var. Qiantouchun and often appear together on the host. When ecological opportunities abound, some phenotypic traits influencing the use of the environment may be differentiated by natural selection, and this can lead to reduced gene flow between populations, and, in some cases, initiate the formation of new species [22]. Alternatively, speciation is driven by mechanisms that have little to do with differences in environmental or ecological opportunities. Phenotypic differences may occur independently of this process, or may occur later, may be caused by interspecific competition or other interactions [2, 23]. In a previous study, we have known these two weevils coexist on a single host, there were significant differences in the oviposition sites. *E. scrobiculatus* females laid eggs in the soil near *A. altissima* and leaf petioles, while *E. brandti* females laid eggs in the trunk of *A. altissima *[24]. The eggs laid by *E. scrobiculatus* in the soil were mainly concentrated in the soil at a distance of 0–40 cm from *A. altissima*, in the compound leaf petioles did not exist alone, usually, 3–8 eggs can be found on a petiole of compound leaves. However, the eggs laid by *E. brandti* may be found at any height between 5 and 405 cm in the phloem of the trunk of *A. altissima *[24]. Besides, through behavioral observations, we found that they must first excavate an oviposition cavity before they lay eggs, and excavating must use their rostra [24, 25]. Before this, we have made the following speculation by studying the oviposition sites and oviposition preference of two species of weevils: The difference in sites for egg-laying between *E.scrobiculatus* and *E.brandti* may be caused by the adaptation of their rostra to the ecological properties of the oviposition sites, and the morphology of their rostra may be related to the hardness of the oviposition substrate surface [24]. Therefore, in this study, we hypothesize that these two species of weevils formed different rostrum-type structural during evolution to adapt to behavioral and ecological needs (i.e. laying eggs in different sites). In order to prove this hypothesis, we compared the internal and external rostra morphological structure of these two species in detail. By this comparison, we tried to study the relationship between the oviposition sites and morphological characteristics of the rostra of these studied weevils, and explain why the two weevils can coexist on the same host *A.altissima*.

## Results

### External morphology of rostra

**Fig. 1:**
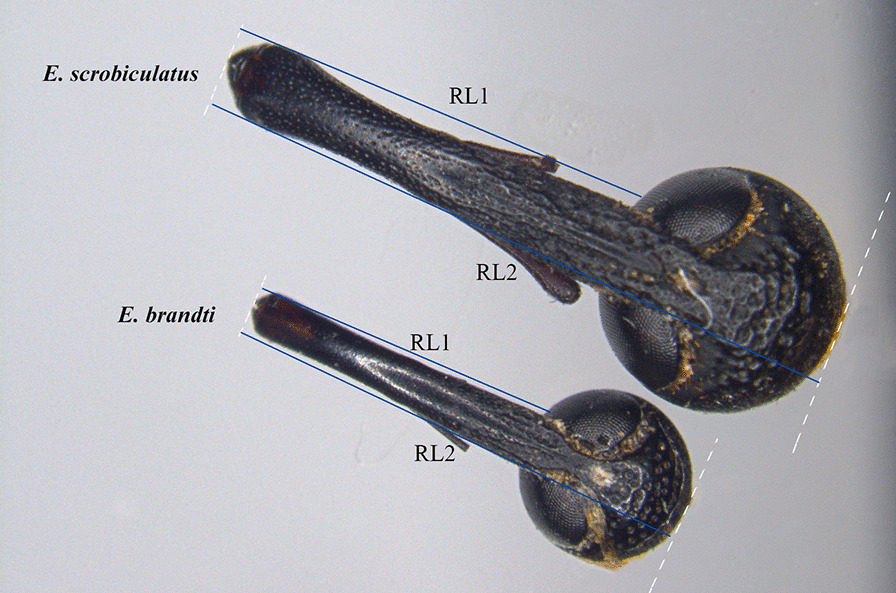
External morphology of rostra of *Eucryptorrhynchus scrobiculatus* female and *E. brandti* female (RL1: Length of rostrum but excluding head capsule; RL2: length of the rostrum including head capsule)

As in all weevils, the rostrum of *E. scrobiculatus* and *E. brandti* is an extension of the forehead of the head, which is slightly curved and has mouthparts at the top (Fig. 1). From the overall appearance, the external structure of the rostrum of *E. scrobiculatus E. brandti* was similar, but the latter was significantly smaller than the former (RL1:4.46 ± 0.03mm vs. 3.14 ± 0.03mm, F = 2.735, *P* < 0.001, RL2:6.42 ± 0.03mm vs. 4.58 ± 0.03mm, F = 0.18, *P* < 0.001). Here, in order to ensure the accuracy of the data, we used two indexes to represent the rostrum length (RL1: Length of rostrum but excluding head capsule; RL2: length of the rostrum including head capsule), because there may be errors caused by artificial removal of the head capsule during the experimental operation. The rostrum of *E. scrobiculatus* female was thicker, and the surface of the rostrum was covered with setae and small pores, while the female of *E. brandti* had a thin and smooth rostrum, with fewer setae and pores on the surface.

### The allometric relationship between rostrum length and body size

**Fig. 2:**
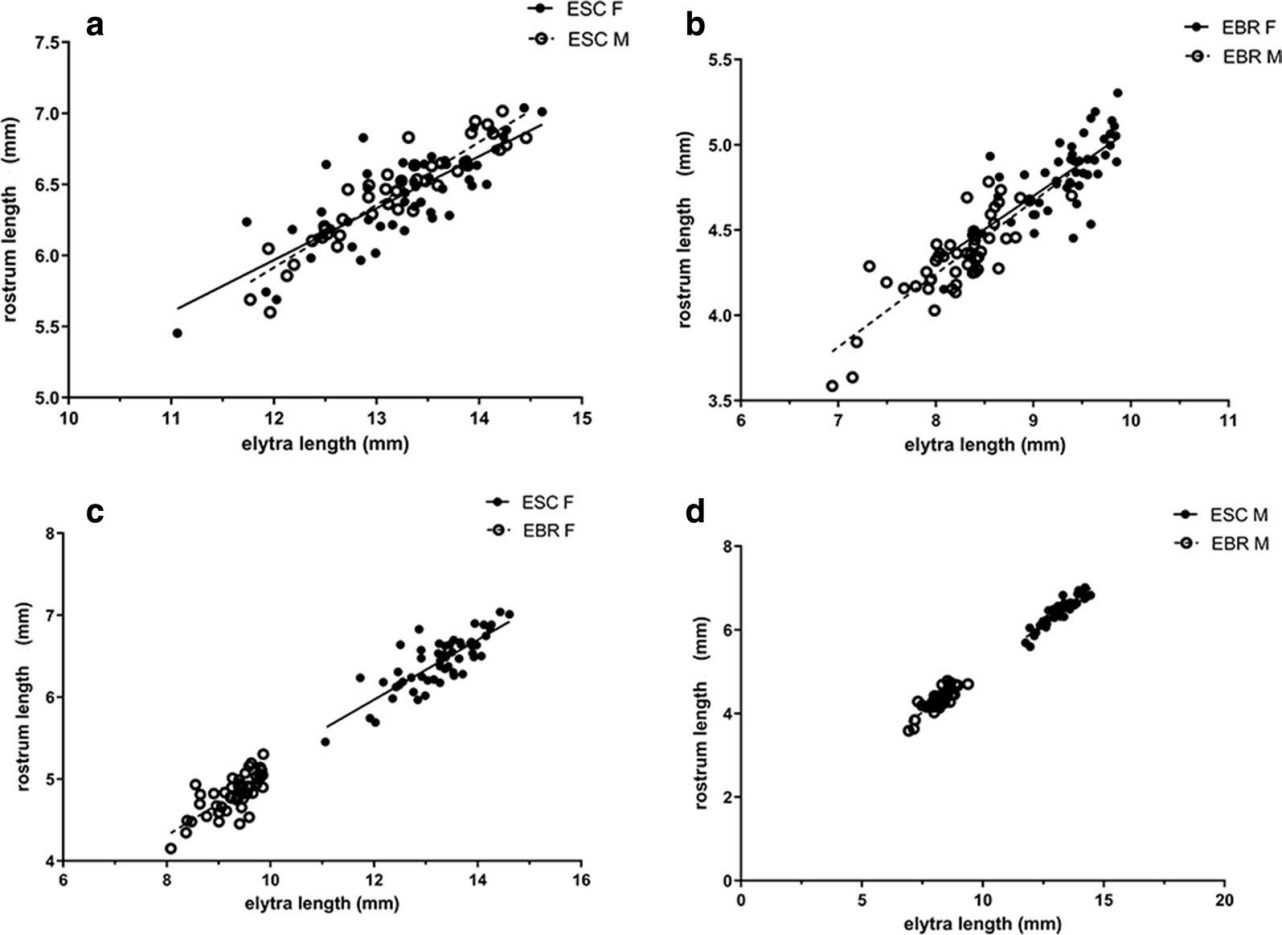
The allometric relationship between elytra length and rostrum length. **a** Relationship between elytra length and rostrum length in *E. scrobiculatus* female and *E. scrobiculatus* male; **b** Relationship between elytra length and rostrum length in *E. brandti* female and *E. brandti* male; **c** Relationship between elytra length and rostrum length in *E. scrobiculatus* female and *E. brandti* female; **d** Relationship between elytra length and rostrum length in *E. scrobiculatus* male and *E. brandti* male

We made a comparison from two aspects: (1) Different sexes of the same weevil. For *E. scrobiculatus*, the test of the allometric relationship between rostrum length and of body size of *E. scrobiculatus* revealed no differences between the sexes (b = 0.820, *P* = 0.112) (Fig. 2a, Table 1). The ANCOVA confirmed (Table 1) the rostrum length was strongly and positively associated with body size (*P* < 0.001), but not with sexes (*P* = 0.278). For *E. brandti*, the test of the allometric relationship between rostrum length and of body size of *E. brandti* revealed no differences between the sexes (b = 0.792, *P* = 0.558) (Fig. 2b; Table 1). The ANCOVA confirmed (Table 1) the rostrum length was strongly and positively associated with body size (*P* < 0.001), but not with sexes (*P* = 0.465). (2) Different species of the same sex. For females, the test of the allometric relationship between rostrum length and of body size of *E. scrobiculatus* females *and E. brandti* females revealed no differences for both female species (b = 0.750, *P* = 0.970) (Fig. 2c, Table 1). The ANCOVA confirmed (Table 1) the rostrum length was strongly and positively associated with body size (*P* < 0.001), but not with species (*P* = 0.314). For males, the test of the allometric relationship between rostrum length and of body size of *E. scrobiculatus* males *and E.bandti* males revealed no differences for both male species (b = 0.861, *P* = 0.393) (Fig. 2d; Table 1). The ANCOVA confirmed (Table 1) the rostrum length was strongly and positively associated with body size (*P* < 0.001), but not with species (*P* = 0.651). In summary, these results indicated that rostrum length of *E. scrobiculatus* and *E. brandti* was determined by body size regardless of sexes and species.

**Table 1:** The allometric relationship between rostrum length and body size in *Eucryptorrhynchus scrobiculatus and E. brandti*

	SEX	95 % CI	F	*P*	ANCOVA (rostrum and body size)	ANCOVA (rostrum and sex)
*E. scrobiculatus*	Female	0.72–0.92	F_1,92_ = 2.581	0.112	F = 270.061	F = 1.189
	Male				*P* < 0.001	*P* = 0.278
*E. brandti*	Female	0.67–0.92	F_1,96_ = 0.346	0.558	F = 161.012	F = 0.538
	Male				*P* < 0.001	*P* = 0.465
*E. scrobiculatus*	Female	0.63–0.87	F_1,100_ = 0.0001	0.970	F = 157.197	F = 1.026
*E. brandti*					*P* < 0.001	*P* = 0.314
*E. scrobiculatus*	Male	0.76–0.96	F_1,88_ = 0.737	0.393	F = 273.489	F = 0.206
*E. brandti*					*P* < 0.001	*P* = 0.651

### Scanning electron microscopy

**Fig. 3:**
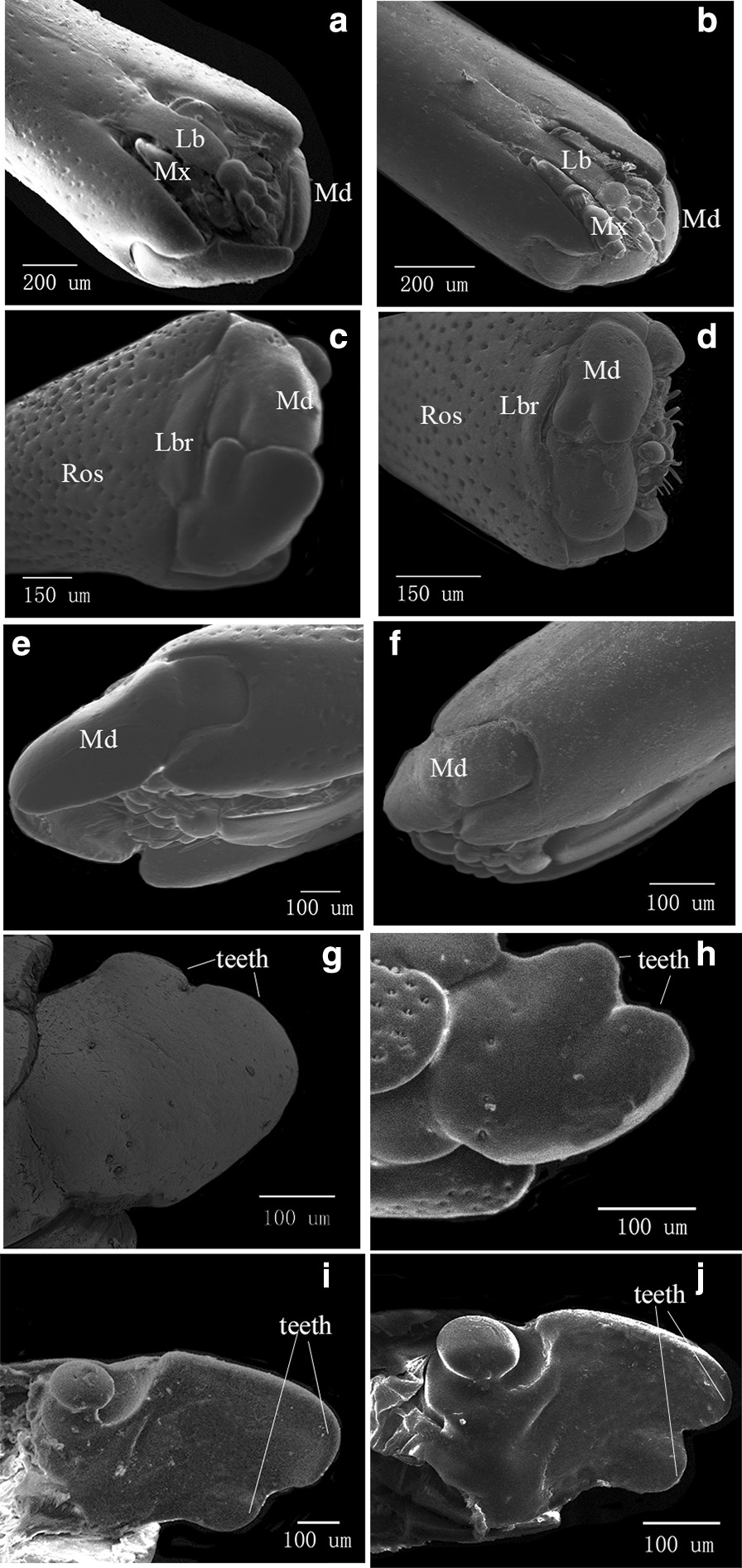
Scanning electron micrographs showing rostra and mouthparts of *E.scrobiculatus* and *E. brandti* females. Mouthparts consist of a labrum (Lbr), a pair of mandibles (Md), a pair of maxillae (Mx) and a labium (Lb). **a** ventral view of *E. scrobiculatus* female mouthpart, which is situated at the apex of the rostrum (Ros); **b** ventral view of *E. brandti* female mouthpart showing obvious differences to that of *E. scrobiculatus* female; **c** apical view of *E. scrobiculatus* female mouthpart; **d** apical view of *E. brandti* female mouthpart; **e** lateral view of *E. scrobiculatus* female mouthpart; **f** lateral view of *E. brandti* female mouthpart; **g** dorsal view of *E. scrobiculatus* right mandible; **h** dorsal view of *E. brandti* right mandible; **i** ventral view of *E. scrobiculatus* right mandible; **j** ventral view of *E. brandti* right mandible

Comparing the detailed structures of female *E. scrobiculatus* (Fig. 3a, c, e, g, i) and female *E. brandti* (Fig. 3b, d, f, h, j), the biggest difference was in the mouthparts. The maxillae and labium of *E. scrobiculatus* were shorter than the mandibles and encased by the mandibles, while the maxillae and labium of *E. brandti* were almost as long as the mandibles and not encased by mandibles completely (Fig. 3a, b). The mandibles of both weevils were massive and irregular hemispherical, the inner surface of each mandible was roughly plane, while the outer surface was strongly convex (Fig. 3e, f). There were prominent teeth on the anterior edge of mandibles, and differences in the form and extent of these teeth result in the mandibles being markedly asymmetrical. Each mandible of *E.scrobiculatus* possessed two teeth, a conspicuous large apical tooth and a small tooth, these two teeth differ greatly in size (Fig. 3c, g, i). Each mandible of *E. brandti* also had two teeth, but the difference in size between the two teeth was not very obvious, almost each one occupied half of the top of the mandible (Fig. 3d, h, j).

### X-ray microtomography and 3D model reconstruction

**Fig. 4:**
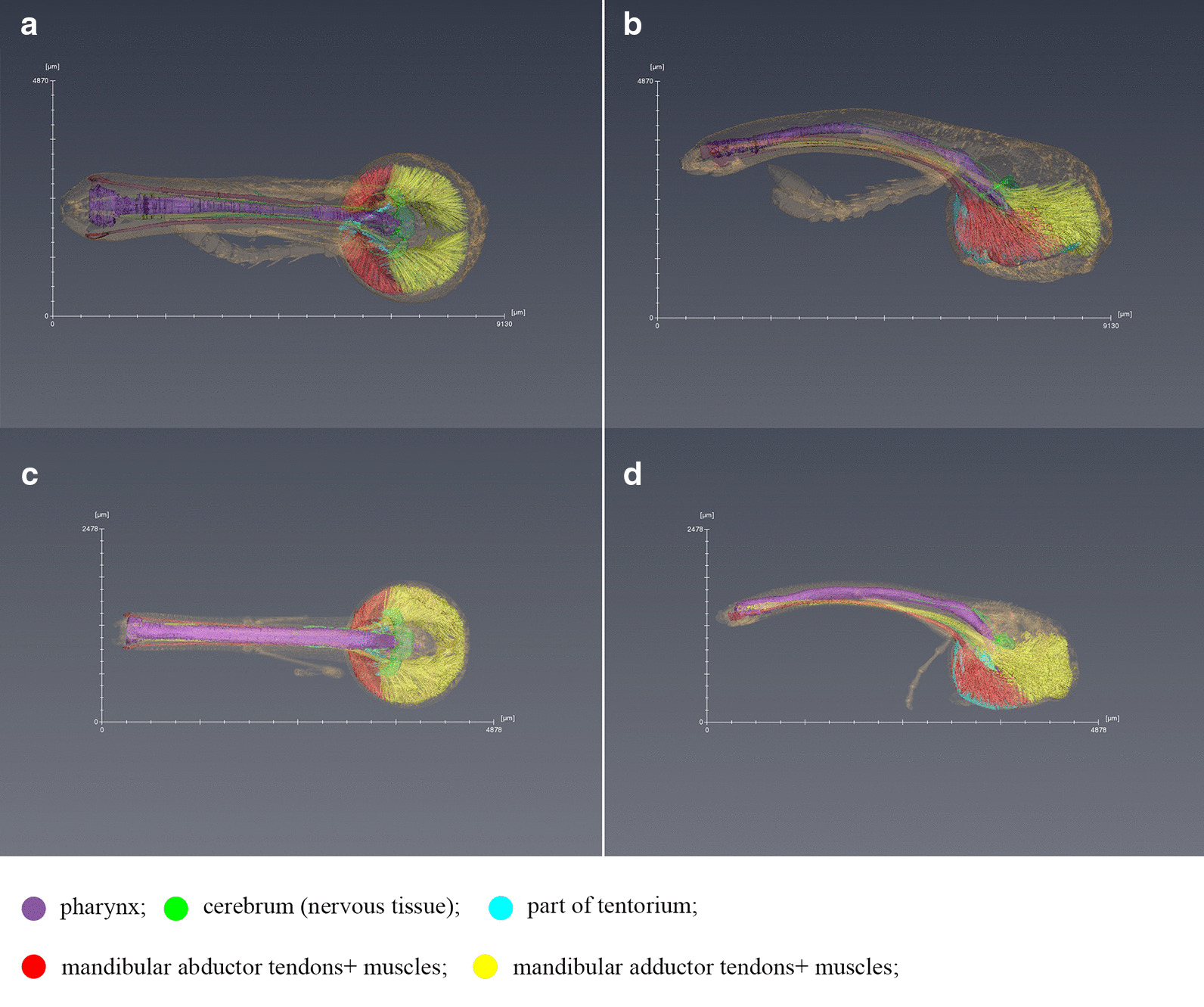
Micro-CT scan of *E.scrobiculatus* and *E. brandti* females, adult head, cuticle of head rendered semitransparent to reveal internal anatomy. **a** Anterior view of *E. scrobiculatus* female; **b** Left side view of *E. scrobiculatus* female; **c** Anterior view of *E. brandti* female; **d** Left side view of *E. brandti* female

Using three-dimensional reconstruction technology, we constructed the two species of weevil’s internal structures, as shown in Fig. 4, the references for the definition and naming of each part are as follows [8, 26, 27]. There is no big difference in the basic internal structure of the two weevils.

**Fig. 5:**
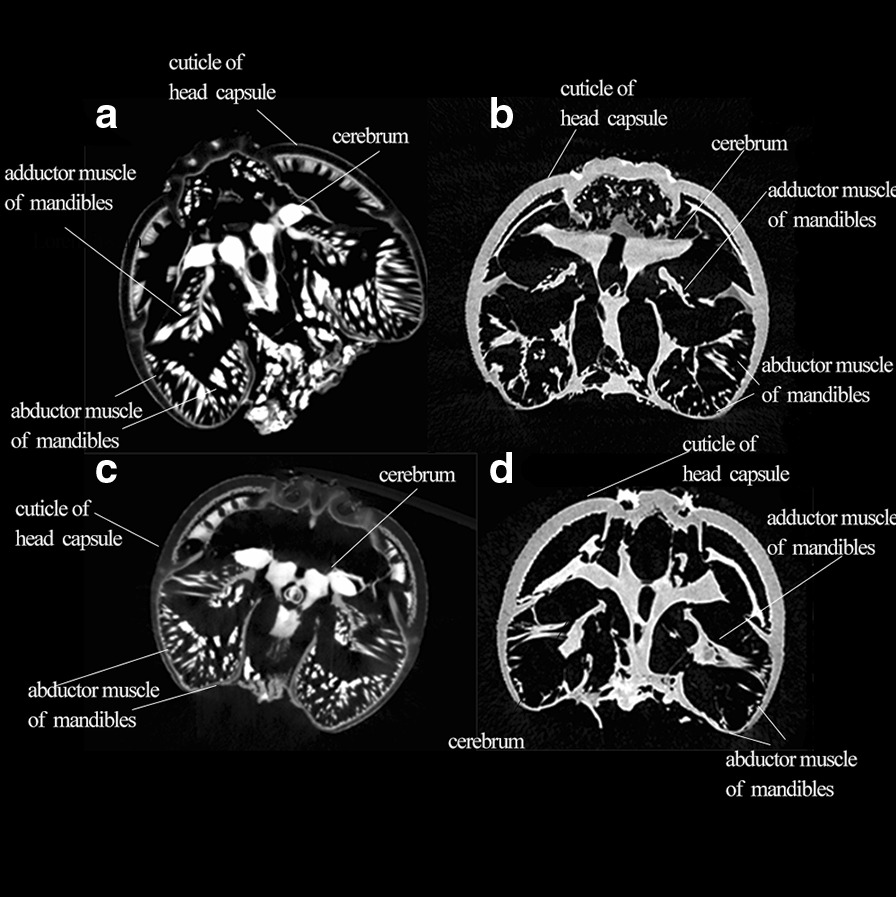
Micro-CT slices of **a**, female *E. scrobiculatus*; **b**, male *E. scrobiculatus*; **c**, female *E. brandti*; **d**, male *E. brandti*

A comparison of the internal male and female mandibular muscles of *E. scrobiculatus* and *E. brandti* revealed a significant difference. It can be seen on the slice of the micro-CT, whether *E. scrobiculatus* or *E. brandti*, the female muscles (mandible adductor muscle and mandible abductor muscle) are more developed than the male (Fig. 5). Based on the construction of the internal structure of the weevil’s rostrum and head capsule, we compared the size of the mandibular muscles volume between males and females of the same species. The results show that (Table 2) the females muscle volume (mandible adductor muscle and mandible abductor muscle) was much larger than that of males regardless of *E. scrobiculatus* and *E. brandti* (*P* < 0.05). Although fixation and dehydration may cause significant shrinkage of muscle tissue so that these volumes cannot represent the absolute volume of living animals, accurate relative comparisons can still be made.

**Table 2:** Comparison of the mandibular muscles volume between males and females of *Eucryptorrhynchus scrobiculatus and E. brandti*

	*E. scrobiculatus* (F)	*E. scrobiculatus* (M)	*E. brandti* (F)	*E. brandti* (M)
V_ad_	5.42 %	3.76 %	6.55 %	4.34 %
V_ab_	3.20 %	1.90 %	2.7 %	1.48 %

V_ad_ = the muscle volume of mandible adductor; V_ab_ = the muscle volume of mandible abductor

**Fig. 6:**
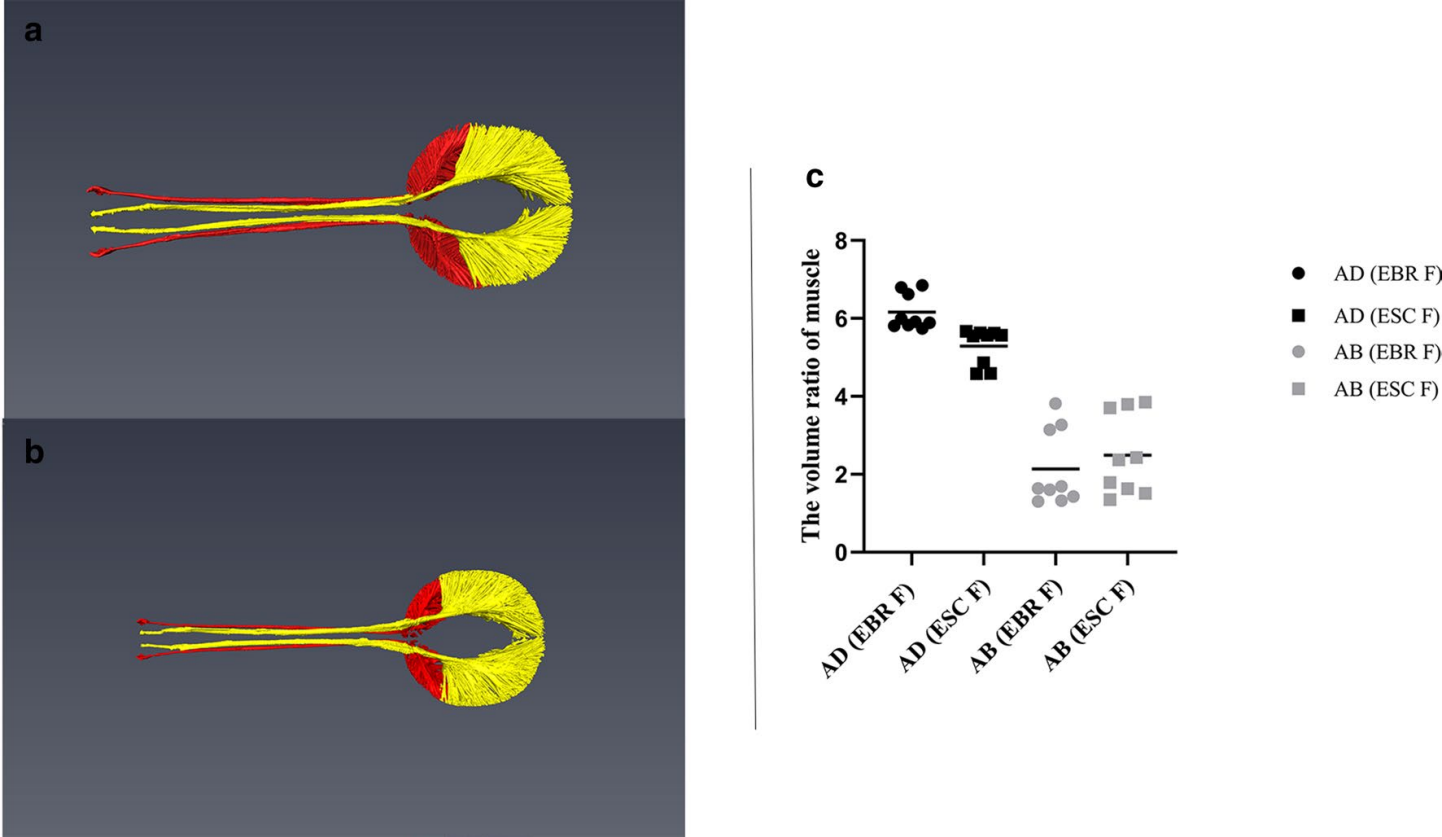
Mandibular muscles of *E. scrobiculatus* females and *E. brandti* females. Pictures **a** and **b** are three-dimensional reconstruction of the mandibular muscles of two kinds of weevils. **a** Mandibular muscles of *E. scrobiculatus* females, the red part represents the abductor muscles, the yellow part represents the adductor muscles; **b** Mandibular muscles of *E. brandti * females, the red part represents the abductor muscles, the yellow part represents the adductor muscles. The picture **c** is the volume ratio of mandibular muscles of *E. scrobiculatus* females (ESC F) and *E. brandti* females (EBR F), AD is the adductor muscle, AB is the abductor muscle

Furthermore, based on the construction of the internal structure of the weevil’s rostrum and head capsule, we compared the size of the mandibular muscles volume of *E. scrobiculatus* females and *E. brandti* females. The results showed (Fig. 6) that the adductor muscle of *E. scrobiculatus* females occupying 5.29% of the head volume was smaller than that of *E.brandti* females occupying 6.16% (F = 15.838, *P* = 0.016 < 0.05), while there was no significant difference between the two types of weevil abductor volume (ES 2.49% VS EB 2.1% F = 0.542, *P* = 0.502 > 0.05). But overall, no matter whether it was *E. scrobiculatus* females or *E. brandti* females, the adductor muscle was significantly larger than the abductor muscle (EB: F = 131.853, *P* < 0.05; ES: F = 45.370, *P* = 0.003 < 0.05). Although fixation and dehydration may cause significant shrinkage of muscle tissue so that these volumes cannot represent the absolute volume of living animals, accurate relative comparisons can still be made.

## Discussion

It has reported that the genetic distance between *E. scrobiculatus* and *E. brandti* was 0.173, the differentiation time of the two weevils can be traced back to about 3.76 million years ago. The evidence suggests these two species may descend from a common ancestor [28]. So far, Liu has studied the phylogeny and differentiation of these two weevils  [28]. He argued that for these two weevils, climate change can lead to a decrease in host resources, which increases competition within populations and leads to differentiation of niches. For this reason, groups occupying different niches may have barriers to gene exchange, and new species are formed through reproductive isolation [28]. In this long-term evolution process, they have formed into different forms to meet different ecological demands. In the previous study we have known, in the long-term evolution process, *E. scrobiculatus* and *E. brandti* utilized different oviposition sites to facilitate coexistence on the single host *A. altissima*. Further observation of oviposition behavior revealed that females must dig a oviposition hole with their rostra before laying eggs [24, 25]. Therefore, before this study we put forward the hypothesis that the difference in the egg-laying positions of the two kinds of weevils may be related to the difference in the structure of their rostra.

In Curculionidae, it is well documented exaggerated rostra of females, and the length of the female’s rostra is considered to be a response to oviposition sites [4, 26, 29–31]. For example, in *Rhopalapion longirostre* (Coleoptera: Brentidae: Apioninae), the rostrum of females was twice as long as males. The elongated rostrum structure of the female *R. longirostre* was a response to bore the maximum depth of the egg channel in the host plant buds [8]. A series of studies show in great detail that the length of the female rostrum is closely related to the relative thickness of the pericarp of the host plant *Camellia japonica* in different populations of the weevil species *Curculio camelliae* (Curculionidae) [3–5]. The *Antliarhinus zamiae* female uses its extremely long rostrum to bore the cone cell of its host plant (Zamiaceae) and lays its egg into the ovule of the cycad [30, 32]. But there are some special examples. There are two species of weevils (large *Curculio elephas* and small *C. glandium*) co-occur in the oak forest. The results showed that there was no difference in the allometric relationship between body size and the rostrum length of the two species females, and the rostrum length was equally correlated with the body size between them [33]. For increased rostrum length, it is likely to be a byproduct of larger individuals in bigger acorns [33]. Our results also prove this, in our study, there was no divergence in adult allometry between the same species of different sexes and same sex of different species of *E. scrobiculatus* and *E. brandti* (Fig. 2). Therefore, we speculate that the rostrum length of *E. scrobiculatus* and *E. brandti* which was caused by body size was not a feature of the two weevil’s adaptation to the oviposition site, it was probably a by-product of the larger body sizes. Perhaps the co-vary in body features and exaggerated feeding traits, rather than just exaggerated rostrum length, is one of the performances of the two kinds of weevil’s adaptation to the environment, this needs further exploration.

Besides, we know the morphological structure of the rostrum plays an important role in the oviposition process of weevil. It is speculated that species of the genus Curculio attacked host plants widely, which is the result of ecological morphological adaptation to the oviposition site, and it is speculated that the size of the seed is the cause of the morphological change in the size of the rostrum [2]. In Belidae and the rest of other weevil families, by fusing the labrum and clypeus and developing advanced mandibles with long pharyngeal processes, the rostrum can be transformed into a suitable oviposition tool so that their eggs can be deposited in firm plant tissues and their larvae truly develop endogenously  [34]. In Attelabidae, rostra of the females are further modified from the belid condition by fusing the gular sutures and reducing the ligula. Females use this rostrum structure to prepare their oviposition sites [34]. For *Rhopalapion longirostre*, the female rostrum has a smooth surface and is suitable for drilling a long borehole through the thick sepals, while contrast to the female long and smooth rostrum, the male rostrum lacks these adaptive structures and cannot bore into the bud tissue deeply [8]. It was argued that natural selection drives a co-evolutionary arms race between weevils and their host plants and that specific morphological changes in curculio’s rostrum result from ecological morphological adaptation to oviposition sites.

In our study, the gross morphological characteristics of the mouthparts of *E. scrobiculatus and E. brandti* are similar to those reported for other weevils [31, 35–39], but their detailed structure is slightly different. Highly sclerotic left and right mandibles are massive or irregular hemispherical, hinged to the later apical margin of the rostrum through developed dorsal and ventral joints, and converge medially. The asymmetrical mandibles look like palm-shaped pliers, the outer surface protrudes in an arc shape. The left and the right mandibles move more or less obliquely upwards and gather towards the rostrum when retracted. Each mandible of *E.scrobiculatus* female possesses two teeth, a conspicuous large apical tooth and a small tooth, these two teeth differ greatly in size (Fig. 3c, g, i). The bigger tooth is round, blunt, and wedge-shaped. According to the principle of ground mechanics, wedge structure can not only reduce the stress concentration but also enhance the mechanical strength of the biological material to adapt to the environmental conditions and improve the wear resistance under the action of soil abrasives. It can also improve the distribution of soil stress at the end, change the shape of the compacted soil, and reduce soil adhesion [40]. In addition, compared with *E. brandti*, the *E. scrobiculatus*’s rostrum has convex edges, grooves and many setae and pores on the whole surface (Fig. 1). Such obviously non-smooth surface not only makes a certain gap between the rostrum surface and the soil surface, reduces the actual contact area of both, and reduces the pressure of air on the interface, but also makes the water film that generates adhesion force between the rostrum surface and the soil interface discontinuous, thereby reducing the soil adhesion to the rostrum surface. Furthermore, due to the elastic deformation and micro-vibration of many setae under the pressure of the soil and the relative motion of the unsmooth rostrum surface, the soil is not easy to adhere to the rostrum surface. Each mandible of *E. brandti* female also has two teeth, but the difference in size between the two teeth is not obvious, almost either one occupies half of the top of the mandible (Fig. 3d, h, j). Based on the observation of oviposition behavior of *E. brandti*, we speculated that two teeth with little difference in their mouthparts were more conducive to mutual friction, tearing and biting bark. In addition, Gertha argued that the smooth surface of the rostrum and the mouthparts at the apex are all good tools for drilling a long hole in the host plants [8]. The overall rostrum surface of the *E. brandti* female is thin and smooth, and there are fewer setae and pores, so it is more conducive to drilling on the trunk.

The mandibular muscles, the adductors and abductors, work together to control the movement of mandibles. Previous studies on the oviposition behavior of the two species have revealed that during the oviposition process, the mandibles of the female were continuously opened and closed to excavate an oval oviposition cavity hole at the oviposition substrate [24, 25], and the mandibles was jointly controlled by the adductor and abductor. The adductor muscle controls the mandibular occlusion, and the abductor muscle controls the mandibular extension [27]. The results showed that whether *E. scrobiculatus* and *E. brandti*, females mandibular adductor muscle and females mandibular abductor muscle were significantly larger than that of males (Table 2). This result is consistent with previous work comparing the mandibular muscles of *R. longirostre* males and females, where females mandibular muscle was found to be significantly larger than that of males  [8]. This difference between males and females’ muscles indicates that females need more muscle to increase muscle strength for excavating oviposition sites, and the muscle strength of females is an adaptation to boring an oviposition hole. In addition, we have known *E. scrobiculatus* females had no difference in abductor muscles than *E. brandti* females, but the adductor muscles of *E. scrobiculatus* females were smaller than that of *E. brandti* females. From this, we can speculate that there was no difference in the amount of external tension between *E. scrobiculatus* females and *E. brandti*, but the bite force on the mandibular of *E. brandti* females was larger than that of *E. scrobiculatus* females, this difference makes *E. brandti* females more suitable for biting the bark and laying eggs on the trunk.

## Conclusions

Different structures determine different functions. In this study, we have compared the internal and external structures morphology of rostrum and head of *Eucryptorrhynhus brandti* and *E. scrobiculatus* in detail. The results are consistent with our hypothesis that there are some differences in the rostra of the two weevil species to adapt to different oviposition sites, irrespective of rostrum length. These two species of weevils show structural differences that reflect the functional potential ovipositional tactics of rostra in the outer and inner morphology to adapt to ecological demands of egg deposition, these results also provide new insights into the coexistence of two weevil species in the same host *A. altissima*. In addition to pest control, the comparison of the two weevil rostra structures provides guidelines for the design of oviposition substrates for the large-scale indoor breeding of the two weevils, as well as provided new inspiration for the prevention and control of these two weevils in the field from the perspective of changing the insect oviposition substrate.

## Material and method

### Study objects

*Eucryptorrhynchus scrobiculatus* adults and *E. brandti* adults were collected from an *A. altissima* forest in Xiaoxingdun village (38°51’N, 106°31’E), Ningxia Hui Autonomous, China, from July 2018 to September 2019.

### External morphology data

The outside micrometer (Guanglu 211-101E, measuring range 0-25mm, Resolution of 0.001 mm) was used to collect the external morphology data of *E. scrobiculatus* (54 female, 43 male) and *E. brandti* (51 female, 52 male). Rostrum length and elytra length of the two species weevils were measured. Rostrum images of *E. scrobiculatus* and *E. brandti* females were taken with a Leica M205FA stereomicroscope (Leica Microsystems).

### Scanning electron microscopy

Seven females of *E. scrobiculatus* and *E. brandti* were fixed in 70% ethanol. The rostrum with mouthparts was first removed from the body with fine forceps and needle. Then the samples were fixed in 2.5% glutaraldehyde for two hours and washed with phosphate buffer at pH7.8 three times for 15 min. The samples were then dehydrated in a graded alcohol series of 30%, 50%, 70%, 80%, 90%, 95%, 100%, in each case for 15 min, and with one repeat at 100% ethanol. The 100% ethanol was then replaced with 100% tertbutanol in a graded series (3:1, 2:2, and 1:3, by volume) for 15 min at each step. Specimens were stored in 100% tertbutanol for 30 min. The specimens were critical point-dried, mounted on stubs with double-side sticky tape, and were sputter-coated with gold by an E-1010 sputter ion instrument (Hitachi, Tokyo, Japan) before examination with an S-3400 N (Hitachi) scanning electron microscope at an accelerating voltage of 0.5–30 KV.

### X-ray microtomography

Twelve specimens each of *E. scrobiculatus* and *E. brandti* were used in this experiment, nine females and three males. The rostrum with mouthpart was also first removed from the body with fine forceps and needle. Then these samples were dehydrated in alcohol. The steps of gradient dehydration in alcohol were as follows: 50% alcohol, 70% alcohol, 75% alcohol, 80% alcohol, 85% alcohol, 90% alcohol, 100% alcohol (3 times), each interval 10–15 min. Finally, dehydrate with acetone 3 times for 30 min each. The prepared samples were scanned using a 3D X-ray microscope (nanoVoxel-3000, Sanying Precision Instrument Co., Ltd.) The specific steps are as follows: the sample was glued to the carbon fiber rod, clamped with a holder, and placed in a test device for 10 min. Turn on the power switch of the device and enter the appropriate test conditions: 60.0 kV, 80.0µmA, 0.60 s. Turn on dynamic scanning and adjust the rotation center of the sample to ensure that the area measured by the sample was always in the field of view during the test. Select the CT test to start scanning. After the scan is completed, transfer the data collected by the device to the back-end machine and import the analysis software.

### 3D model reconstruction

The imported data was analyzed with Avizo 9.0.1 to reconstruct the target structure. Morphological structures were segmented manually using mainly the brush, lasso, magic wand tool of the Avizo Segmentation editor. The main structures inside the weevils’ head were divided and extracted from the three angles of the test sample XY, XZ, and YZ axes. Different structures are marked in different colors and combined in the project view of Avizo and were trimmed and smoothed with volume edit. Finally, solid and transparent surfaces were rendered using multiple viewers. The internal space of the entire head (including the head capsule and rostrum) was filled and the antennae were removed using the volume editing tool as the denominator of the volume ratio analysis. Take the segmented muscle volume as the input image, the head fill and remove antennal as input image mask by volume fraction of Avizo to calculate volumes proportion of the muscle.

### Statistical analyses

The rostrum length and body size (elytra length) between the adult of *E.scrobiculatus* and *E.brandti* were compared through ANOVA (Analysis of Variance). The method of Toju and Sota [4] for intraspecific population analyses was used to compare different sexes of the same weevils and different species of the same sex, to detect the difference in the proportional relationship between adult rostrum length and body size. In this method, the function y = ax^b^ was used to evaluate allometry, where x and y correspond to the body size and rostrum length, respectively. The log-transformed was log y = log a + blog x, and therefore the slope “b” was calculated to evaluate the allometric relationship between body size and rostrum length. On this basis, we conduct ANCOVA (Analysis of Covariance), in which the rostrum length of logarithmic transformation was a dependent variable, species or sexes was an independent factor, and the body size was a covariate. We evaluated whether the allometric relationship of rostrum length and body size ( slope of function ) between species or sexes was different and whether the influence of species/sexes on the length of the rostrum was independent. In addition, we used a one-way ANOVA method to compare the difference in rostra length and muscles volume between the two weevils.

## Supplementary Information

**Additional file 1: Fig. S1.** The excavating behavior of the two species of weevils before depositing eggs. A, the behavior of excavating an oviposition cavity of *Eucryptorrhynchus scrobiculatus*; B, the behavior of excavating an oviposition cavity of *E. brandti*. Usually, during this process, the male lays on the back of the female.

**Additional file 2.** Data of elytra length and rostra length of *Eucryptorrhynchus scrobiculatus* and *E. brandti*.

## Data Availability

The datasets related to the inside of the rostrum taken by micro-CT and 3D model reconstruction are not publicly available due some of our projects are still in progress but are available from the corresponding author on reasonable request. In addition, all other data generated or analysed during this study are included in this published article and its Additional files 1, 2.

## Abbreviations

ES: *E. scrobiculatus*
EB: *E. brandti*
V_ad_: The muscle volume of mandible adductor
V_ab_: The muscle volume of mandible abductor
