# Supplementary material for: Structural comparison of the rostra of two species of weevils coexisting on Ailanthus altissima: the response to ecological demands of egg deposition
Source: BMC Ecol Evol. 2021 May 28;21:101. doi: 10.1186/s12862-021-01824-7 (PMC8161989; doi:10.1186/s12862-021-01824-7)
Supplement: Supplementary file 1 — Additional file 1: Fig. S1. The excavating behavior of the two species of weevils before depositing eggs. A, the behavior of excavating an oviposition cavity of Eucryptorrhynchus scrobiculatus; B, the behavior of excavating an oviposition cavity of E. brandti. Usually, during this process, the male lays on the back of the female. [file 12862_2021_1824_MOESM1_ESM.docx]

**Figure S1.**


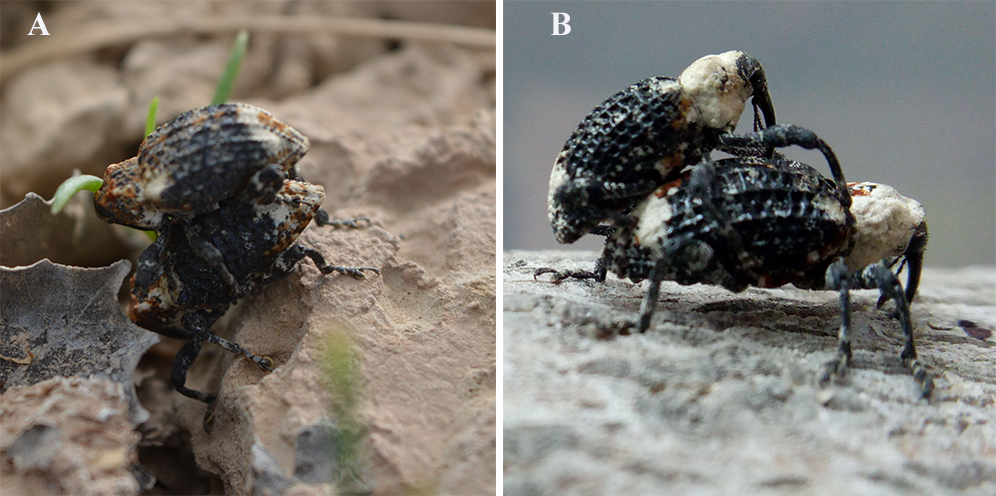


**Figure S1.** The excavating behavior of the two species of weevils before depositing eggs in the field, the picture on the left is *E. scrobiculatus*, the picture on the right is *E. brandti.* Usually, during this process, the male lays on the back of the female.
